# Salt-Induced Stress Impacts the Phytochemical Composition and Aromatic Profile of Three Types of Basil in a Genotype-Dependent Mode

**DOI:** 10.3390/plants12112167

**Published:** 2023-05-30

**Authors:** Michele Ciriello, Valerio Cirillo, Luigi Formisano, Stefania De Pascale, Raffaele Romano, Giovanna Marta Fusco, Rosalinda Nicastro, Petronia Carillo, Marios C. Kyriacou, Georgios A. Soteriou, Youssef Rouphael

**Affiliations:** 1 Department of Agricultural Sciences, University of Naples Federico II, 80055 Portici, Italy; michele.ciriello@unina.it (M.C.); valerio.cirillo@unina.it (V.C.); luigi.formisano3@unina.it (L.F.); depascal@unina.it (S.D.P.); raffaele.romano@unina.it (R.R.); 2 Department of Environmental, Biological and Pharmaceutical Sciences and Technologies, University of Campania “Luigi Vanvitelli”, Via Vivaldi 43, 81100 Caserta, Italy; giovannamarta.fusco@unicampania.it (G.M.F.); rosalinda.nicastro@unicampania.it (R.N.); petronia.carillo@unicampania.it (P.C.); 3 Department of Vegetable Crops, Agricultural Research Institute, Nicosia 1516, Cyprus; mkyriacou@ari.moa.gov.cy (M.C.K.); soteriou@ari.moa.gov.cy (G.A.S.)

**Keywords:** antioxidant activities, aromatic plant, linalool, NaCl, *Ocimum basilicum* L., terpenes

## Abstract

Basil (*Ocimum basilicum* L.) is among the most widely used aromatic plants of Lamiaceae, often grown in areas where salinity is an adverse factor. Most studies on the effect of salinity on basil focused on the influence of salt stress on productive traits, while few reported on how it affects the phytochemical composition and the aroma profile. Three basil cultivars (Dark Opal, Italiano Classico, and Purple Ruffles) were grown hydroponically for 34 days with two nutrient solutions that differed in NaCl concentration [no NaCl (Control) and 60 mM NaCl]. Yield, secondary metabolite concentration (β-carotene and lutein), antioxidant activity [1,1-diphenyl-2-picrylhydrazyl (DPPH) and ferric reduction antioxidant power (FRAP)], and aroma profile based on composition of volatile organic compounds (VOCs) were appraised in response to salinity applications. Salt stress significantly reduced fresh yield in Italiano Classico and Dark Opal by 43.34 and 31.69%, respectively, while no effect was observed in Purple Ruffles. Furthermore, the salt-stress treatment increased β-carotene and lutein concentrations, DPPH, and FRAP activities, and the total nitrogen content of the latter cultivar. CG-MS analysis revealed significant differences in VOCs composition of the basil cultivars, with Italiano Classico and Dark Opal characterized by the predominance of linalool (average 37.52%), which, however, was negatively affected by salinity. In Purple Ruffles, the predominant VOC compound, estragole (79.50%), was not affected by the deleterious effects of NaCl-induced stress.

## 1. Introduction

Universally known as the ‘king of herbs’, basil (*Ocimum basilicum* L.) is the most representative species of the genus *Ocimum* and the iconic aromatic herb of the Lamiaceae family [1]. Cultivated worldwide, it owes its fame to its multifaceted nature, resulting from the genetic diversity that characterizes the genus. About 20 species were selected and bred in recent years [2], and the number of commercial cultivars is steadily increasing due to the ongoing demands of world markets [3]. Although many differences within cultivars relate to morphological aspects (such as leaf color, shape, and size) [4,5], the phytochemical composition remains the crucial discriminant. The different uses this plant provides, ranging from food to pharma-cosmetic, can be attributed to specific secondary metabolites that are increasingly sought after [6,7]. The composition of volatile compounds is undoubtedly the most important and studied aspect of aromatic plants [8]. More than a thousand plant-derived volatile organic molecules were reported, and their knowledge and distribution in plants were greatly improved in recent years through the adoption of increasingly sensitive, simple, and inexpensive analytical methods [9]. Due to their known antioxidant, acaricidal, insecticidal, antifungal, nematocidal, and bactericidal activities, many of these odorous molecules could be successfully exploited by the green industry, which is always looking for natural substitutes for synthetic molecules [10]. The growing interest in products of natural origin, which are readily available and free of side effects, increased the interest in medicinal plants due to their beneficial properties that make them cheap and renewable ingredients for producing natural preservatives, cosmetics, and new types of drugs [11,12]. Currently, in most developed countries, approximately 25% of drugs are of plant origin, while in developing countries, this percentage is much higher, reaching approximately 80% [13]. For each basil genotype, the flavor and aromaticity of its leaves are the result of a cocktail of phenylpropanoids (such as estragole, eugenol, and methyl eugenol) and terpenoids (such as linalool, eucalyptol, trans-α-bergamotene, geranial, neral, β-ocimene, β-caryophyllene, α-cubebene, and γ-muurolene), which are synthetized through specific metabolic pathways (such as shikimate and mevalonate, respectively) and secreted by glandular trichomes on basil leaves [1,14,15]. Despite the aforementioned genetic diversity, most studies were focused on the aromatic characterization of the Genovese type because of its role in Italian gastronomy [16,17]. However, the lack of studies on the influence of individual environmental factors (climate and agronomic practices) and their mutual interactions on the biochemical pathways leading to the specific aromatic profile in basil hindered the knowledge of the genetic imprint on this key qualitative aspect, not only on basil but on all medicinal plants [18,19,20,21,22]. As with most leafy vegetables, basil growth, development, and yield can be affected by environmental factors such as drought, extreme temperatures, and salinity [23]. The latter is one of the most challenging factors in the agricultural sector, being an expanding phenomenon worldwide as a cause of climate change. Generally, as a result of excess NaCl in the rhizosphere, salt stress induce osmotic and toxic effects which damage the plant by limiting normal metabolic functions, thus impairing its growth and yield [24]. Always considered moderately tolerant to salinity [25,26,27,28], basil has great potential as a multipurpose crop in areas where soil salinity poses a problem to grow other crops that are more sensitive to this environmental constraint. In this regard, the high genetic variability of basil could represent an useful tool to limit the harmful effects of salinity [29,30]. Moreover, salt stress could elicit positive responses on the secondary metabolism of basil and leafy vegetables, via the increase in the synthesis of aromatic compounds highly desired by industry and consumers [13,31,32]. In any case, as Jadczak et al. [33] pointed out, the economic evaluation of basil cultivation cannot completely exclude the production aspect. Indeed, although salinity increases the biosynthesis of secondary metabolites (within specific limits), the resulting decrease in fresh biomass could result in their reduction per unit area. In any case, most of the studies in the literature focused on evaluating the effects of salinity on the yield and composition of the basil phenolic profile, neglecting the response of the aromatic profile to the imposition of the stress. In view of these considerations, the purpose of the present work was to evaluate the influence of 60 mM NaCl on the aroma profile, the growth penalty, and the antioxidant activity of three basil cultivars (Dark Opal, Italiano Classico, and Purple Ruffles).

## 2. Results

### 2.1. Yield and Plant Morphology

**Figure 1:**
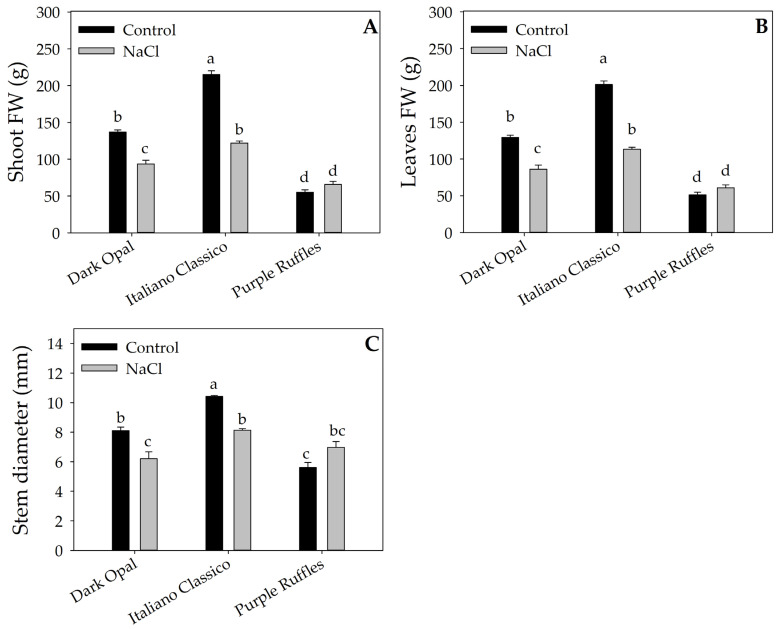
Effects of Cultivar × Stress interaction on shoot FW (**A**), leaves FW (**B**), and stem diameter (**C**). Different letters indicate significant mean differences according to Tukey HSD test (*p* = 0.05).

Production and biometric parameters shown in Table 1 and Figure 1 were found to be significantly affected by Cultivar (CV) and Stress (S) factors and their interaction. Regardless of NaCl treatment, Italiano Classico was characterized by the highest values of shoot fw (168.49 g plant^−1^), leaves fw (157.219 g plant^−1^), and stem diameter (9.274 mm), while the lowest values were obtained from Purple Ruffles. Regarding CV × S interaction, for Dark Opal and Italian Classico, a significant reduction in shoot fw (Figure 1A), leaves fw (Figure 1B), and stem diameter (Figure 1C) was recorded. In contrast, for Purple Ruffles, NaCl treatment (60 mM NaCl) did not result in significant differences compared to the control.

### 2.2. CIELab Colorimetric Parameters

Except for Chroma, all colorimetric parameters shown in Table 2 were significantly affected by the CV × S interaction. Compared with control, the brightness (L) decreased in Dark Opal and Italiano Classico under NaCl treatment, while unchanged in Purple Ruffles. The Greenness index (a*), yellowness index (b*), and Hue angle were significantly affected by NaCl treatment only in Dark Opal. Chroma showed significant genotype-dependent changes, with the highest values recorded in Italiano Classico.

### 2.3. Metabolites and Antioxidant Activities

**Figure 2:**
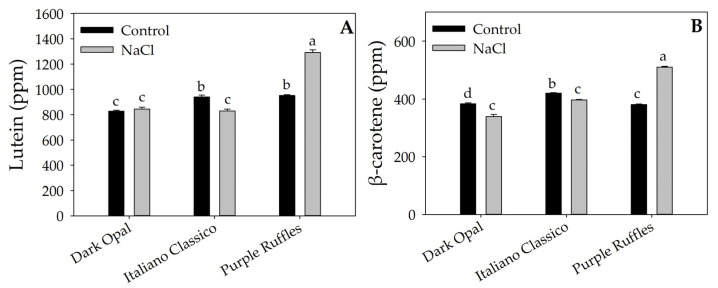
Effects of Cultivar × Stress interaction on leaf concentration of lutein (**A**) and β-carotene (**B**). Different letters indicate significant mean differences according to Tukey HSD test (*p* = 0.05).

**Figure 3:**
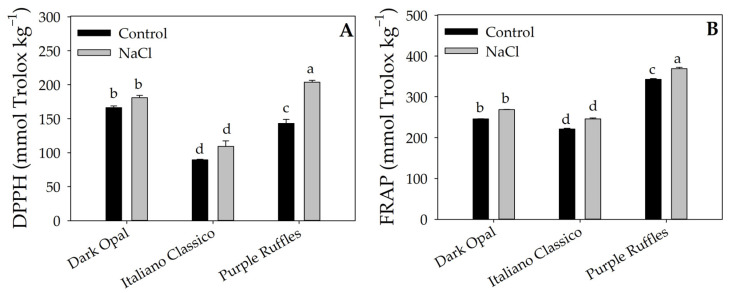
Antioxidant activity measured with DPPH (**A**) and FRAP (**B**) assays of three basil varieties (Dark Opal, Italiano Classico, and Purple Ruffles) grown under control and salt stress conditions. Different letters indicate significant mean differences according to Tukey HSD test (*p* = 0.05).

The factors under investigation significantly influenced lutein and β-carotene concentrations (Table 3) and their interaction (Figure 2). In particular, Purple Ruffles had higher average concentrations of lutein and β-carotene by 30.40 and 15.71%, respectively, compared with the other basil (Table 3). Compared with the control, the Purple Ruffles × NaCl interaction increased lutein and β-carotene concentrations by 35.70 and 33.91%, respectively (Figure 2A,B). An opposite trend was observed for Italiano Classico, where NaCl significantly reduced the concentration of the two carotenoids. Relative to Dark Opal, NaCl treatment did not result in significant differences for lutein, but decreased β-carotene concentration compared with the control. For the DPPH and FRAP antioxidant activities (Figure 3A,B), NaCl did not result in significant differences in Dark Opal and Italiano Classico, unlike Purple Ruffles, in which an increase in their concentration was observed compared to the control.

### 2.4. Aromatic Profile

**Figure 4:**
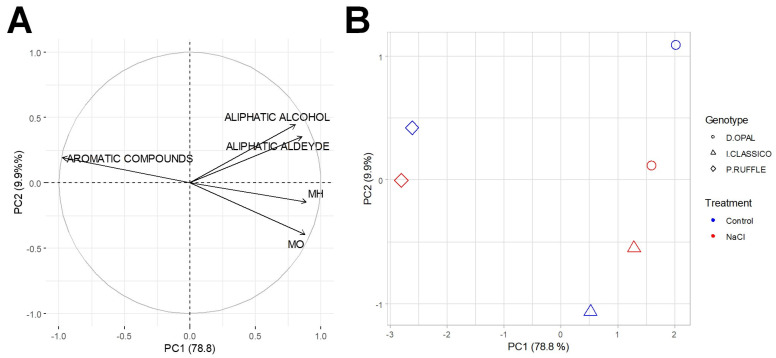
Graphs showing (**A**) variable correlation and (**B**) plotted centroids of three basil varieties (Dark Opal, Italiano Classico, and Purple Ruffles) grown under control and salt stress (NaCl) conditions based on their aromatic profile. The individual coordinates of each replicate were used to calculate group centroids in PC1 and PC2. PC = principal component; MH = Monoterpene hydrocarbons; SH = Sesquiterpene hydrocarbons; MO = Oxygenated monoterpene.

The solid-phase microextraction (SPME)-GC/MS analysis of volatile components was used to determine the flavor profile of three basil varieties (Dark Opal, Italiano Classico, and Purple Ruffles) grown under control and stress conditions. As Figure 4A indicates, the main differences were due to the presence of aromatic compounds, that clearly separate Purple Ruffles from the other two varieties (Dark Opal and Italiano Classico) on the principal component (PC) 1 (Figure 4B). The effect of salt stress was instead more evident on the PC2 that mainly discriminates between aliphatic alcohols/aldehyde and MH/SH/MO (respectively, monoterpene hydrocarbons, sesquiterpene hydrocarbons, and oxygenated monoterpene) (Figure 4A). Salt stress induced minor changes on Purple Ruffles and Italiano Classico varieties on the PC2, while this effect was more evident in Dark Opal (Figure 4B). Specifically, PC1 explained 78.8% of variability, while PC2 explained 9.9% (Figure 4).

Table 4 shows the percentages of the nine significant compounds. Among them, linalool, eucalyptol, and 2-Hexenal accounted for about 75% of the entire flavor profile of Dark Opal and Italiano Classico (on average). In contrast, for Purple Ruffles, about 80% was represented by estragole, a compound absents in the other two varieties. The interaction between the factors considered revealed significant differences for the compounds 2-hexenal, 1-octen-3-ol, eucalyptol, β-cis-ocimene, linalool, and eugenol. Specifically, compared with the control, the Italiano Classico × NaCl interaction increased β-cis-ocimene and 2-hexenal but decreased linalool; a similar trend was observed in Dark Opal. In the latter, a reduction in 1-octen-3-ol was observed in salinized plants compared with that recorded in the control condition. Regardless of salt treatment, Dark Opal, compared with Italiano Classico, had an aroma profile characterized by higher hexanal and eucalyptol but lower eugenol content. In contrast, no significant difference was recorded for trans-α-Bergamotene.

## 3. Discussion

### 3.1. Basil Cultivars Show Different Degrees of Salt Stress Tolerance

Regardless of salt stress, the significant differences recorded between basil cultivars for biometric and yield parameters highlighted the strong effect of the genotype [34]. However, this variability was expected due to the phylogenetic distance and visible morphological differences between cultivars [35]. The red cultivars showed lower shoot fw than the green ones, a phenomenon that can be explained by their constitutively high content of anthocyanins [36]. In fact, the synthesis of these compounds had a high metabolic cost to the plant, which diverts the energy from growth [37]. In addition, it was previously pointed out that anthocyanins act as a light screen, thus reducing the amount of light intercepted by chloroplasts [38]. Therefore, red plants generally show lower dry biomass due to a slower electron transport process through the photosystems, lower production of ATP and NADPH and, consequently, a lower CO_2_ assimilation rate [39].

A further explanation for the improved production performance of the green cultivar could be attributable to the intensive genetic breeding carried out in recent years, as in the case of Italiano Classico. Indeed, this variety is the preferred cultivar for the industrial processing of ‘pesto sauce’ and was improved in its technological and yield characteristics to adapt its cultivation to both open field and soilless production [40]. Our results show that the different basil cultivars had a different degree of salt tolerance, as commonly reported in other species [26,41]. Dark Opal and Italiano Classico significantly reduced shoot fw, probably due to the onset of deleterious osmotic and nutritional stress limiting growth and the uptake of macronutrients such as nitrate, potassium, and calcium [42,43]. Moreover, the cellular accumulation of Na^+^ and Cl^–^ ions beyond the toxicity threshold is a further cause of plant malfunctioning and growth impairment [44].

The reduction in yield parameters followed the same trend as leaves fw and stem diameter; the latter being an important parameter because it is primarily responsible for the mechanical support of the plant, and excessive thinning can cause plant lodging [45], which represents a main problem especially in open field cultivation. Although Dark Opal and Italiano Classico showed a typical response of glycophytic plants in terms of biomass accumulation, Italiano Classico yield loss was much higher that Dark Opal one (Figure 1 and Table 1), indicating a different degree of salt stress tolerance between the two cultivars. Considering that the marketable yield consisting of the entire aboveground biomass is a key parameter for basil, reducing growth due to salinization would be an important limitation on agronomic productivity and economic return.

The different sensitivity to salinity between Italiano Classico and Dark Opal could be attributed to the anthocyanins content or to a more vigorous vegetative growth of Italiano Classico. As argued by Jakovljević et al. [6], a negative correlation between salinity tolerance and growth was often found. This seemed to be in line also with the high tolerance of Purple Ruffles to salt stress, which showed the lowest yield reduction under salt stress, but was also the least productive cultivars under control condition (Figure 1 and Table 1). Indeed, for this cultivar, the concentration of NaCl tested did not affect production and biometric parameters. Different colorimetric responses to salt stress also show a distinct difference between the most sensitive cultivars and the Purple Ruffles (Table 2).

### 3.2. Different Response of Basils Quality Traits to Salt Stress

Color is an essential quality trait for leafy vegetables as it drives consumer and producer choice [16]. Similarly to what Ciriello et al. [41] observed, the leaf CIELab parameters of Dark Opal and Italian Classico were significantly affected by salt treatment. Consistent with what was observed in a similar study [30], in Dark Opal, the leaf brightness increased in the salt treatment, while an opposite trend was observed in Italiano Classico, despite having constitutively higher brightness values. Contrarily, in Italiano Classico, the greenness (a*) did not show significant changes in salt treatment. The presence of shiny leaves that are deep green and homogeneous is a key requirement for the processing industry, as it would reduce the use of colorants in the processing of ‘pesto sauce’.

Other than the macroscopic alterations on yield induced by salinity, this abiotic stress involved more complex biochemical and molecular aspects. Salt stress triggers the production of reactive oxygen species (ROS), which are the primary causes of oxidative damage [46]. To counteract the detrimental effects of ROS on cell membranes, plants enact specific defense mechanisms that generally relies on the generation of secondary metabolites [47]. Bernstein et al. [48] reported that the main effect of salt stress on basil is the change in secondary metabolism. Considering that carotenoids, due to their antioxidant roles, are closely related to the level of tolerance to environmental stressors [23], it is not surprising that the highest lutein and β-carotene were recorded in the most tolerant cultivar (Purple Ruffles), confirming what was observed in *Hordeum vulgare* L. by Khosravinejad et al. [49]. The significant increase in these metabolites in salt stressed Purple Ruffles could be an essential stress-protective mechanism [35]. On the contrary, an opposite trend was observed for Italiano Classico and Dark Opal, where these metabolites were found to be unchanged in response to salt stress (Figure 2 and Table 3).

The higher antioxidant capacity of Purple Ruffles was further confirmed by the results of DPPH and FRAP (Figure 3 and Table 3), in line with the higher concentrations of lutein and β-carotene under salt stress. Interestingly, DPPH and FRAP were significantly higher in Dark Opal and Purple Ruffles leaves compared to Italiano Classico, pointing out the influence of the genotype on plant response to oxidative stress [50].

### 3.3. The Influence of Salt Stress on Basil Aromatic Profile

When discussing aromatic plants, their relationships with the atmosphere are not limited exclusively to gas exchange processes but also with volatile organic compounds, since aromatics invest a significant amount of carbon utilized by these crops [51]. In addition to their important ecological functions (ROS scavenging, tolerance to high temperatures, defense against pathogens and herbivores, and attraction of pollinators) [52], these low-molecular weight compounds have great relevance in the food and pharmaceutical industries, as they can be exploited for numerous biotechnological applications.

The regulation of the biosynthesis of these secondary metabolites is mainly under genetic control [1], but also by environmental cues such as salt stress [53]. The above was confirmed by our data showing significant differences in the aroma profile of basil, irrespective of NaCl stress. Specifically, the aroma profiles of Italiano Classico and Dark Opal were mainly characterized by oxygenated monoterpenes while those of Purple Ruffles by phenylpropanoids (aromatic compounds) (Figure 4). On the other hand, salt stress only marginally affected the aromatic profile of the tolerant variety Purple Ruffles, while its effect was more evident in the sensitive ones Dark Opal and Italiano Classico (Figure 4).

It is possible that the higher stability of the aromatic profile in Purple Ruffles is further evidence of the higher degree of salt stress tolerance, which was mainly due to its highly efficient antioxidant activity (Figure 3). Supporting this hypothesis, the accumulation of specific secondary metabolites acts as a defense mechanism that can help plants adapt to stress [8]. Additionally, this aspect would confirm the negative correlation between the total levels of terpene synthesis and phenylalanine ammonia lyase activities, which are involved in the synthesis of terpenes and phenylpropanoids, respectively [15].

Detailed analysis of the complete aroma profile confirmed that the distinctive aroma of Genovese basil (Italiano Classico) is attributable to the absence of estragole from aromatic molecules such as linalool, eucalyptol, and eugenol [16]. Although in different proportions, these molecules are also characteristic of the aromatic bouquet of Dark Opal, which, however, had a significant amount of 2-hexanal and hexanal (Table 4) that would impart a strong herbaceous scent. Interestingly, after salinization, the latter molecule was found exclusively in Dark Opal and Italiano Classico. This result would confirm the ‘opportunistic hypothesis’ proposed by Peñuelas and Llusià [54] that plants change the demand for essential volatile organic compounds in terms of concentration and composition when responding to environmental stresses.

In partial agreement with this, we observed a reduction in linalool in NaCl-treated Dark Opal and Italiano Classico plants. This result, a consequence of salt stress, could also be attributable to decreased nutrient availability, particularly nitrogen, which is involved in the biosynthesis of primary and secondary metabolites [55]. In addition to the above, the concomitant increase in β-cis-ocimene in salinized Italiano Classico plants could suggest that terpene-synthase improved the biosynthesis of this volatile compound; in fact, a single terpene-synthase can originate several volatile compounds due to the randomness of bond rearrangement [56]. On the contrary, the levels of linalool and estragole in Purple Ruffles, more abundant compounds, were not affected by NaCl stress. Compared to the other cultivars, the subtle changes in the flavor profile of Purple Ruffles suggest that 60 mM NaCl did not result in critical stress levels.

## 4. Materials and Methods

### 4.1. Growth Conditions, Treatments, and Experimental Design

The present experiment was conducted in spring 2021 at the Department of Agriculture of the University of Naples ‘Federico II’, located in Portici (Naples, latitude 40°49′11 ″64 N, longitude 14°20′28″68 E, 29 m above sea level). The cultivation system consisted of an unheated greenhouse (30 m long and 7.5 m wide) irradiated by natural sunlight and with passive ventilation. Basil (*Ocimum basilicum* L.) cultivars Dark Opal (Blumen, Milan, Italy), Italiano Classico (La Semiorto, Sarno, Italy), and Purple Ruffles (Pagano Domenico & Figli Sementi, Scafati, Italy) were transplanted on 29 May 2021, in 1.2 L pots filled with a substrate composed of 2/3 of peat (Vigorplant, Fombio, Italy) and 1/3 of perlite (Perlite Italiana, Corsico, Italy). The plants were arranged in rows (27 × 15 cm) at a density of 25 plants per square meter. The experimental design was bifactorial, in which the factors were three basil cultivars (Dark Opal, Italiano Classico, and Purple Ruffles) and two nutrient solutions (NS; hereafter, Control and NaCl, respectively) differed in NaCl concentration. Specifically, the control NS was a modified Hoagland that had the following micro and macronutrient composition and free of NaCl: 13.0 mM NO_3_-N, 1.0 mM NH_4_-N, 1.5 mM P, 5.0 mM K, 1.75 mM S, 4.5 mM Ca, 2 mM Mg, 9 μM Mn, 20 μM Fe, 0.3 μM Cu, 20 μM B, 1.6 μM Zn, and 0.3 μM Mo. NaCl NS was prepared by adding 60 mM NaCl to the control NS. NSs were delivered through a drip irrigation system with one self-compensating dripper (2 L h^−1^) per plant. Each experimental unit was replicated three times (*n* = 3) and included 20 plants (60 plants per treatment).

### 4.2. Harvesting, Morphological and Yield Measurements, Sample Preparation, and Storage

The experimental trial lasted 34 days (29 May to 2 July). At harvest (34 days after transplanting, DAT), ten representative plants per replicate were cut at the root collar, avoiding border plants, and separated into leaves and stems. Shoot fresh weight (fw; g plant^−1^), leaf fw (g plant^−1^), stem diameter (mm) using a digital caliper (accuracy ± 0.02 mm; RS PRO, Sesto San Giovanni, Milan, Italy). Six plants per replicate were placed in liquid nitrogen, stored at −80 °C, and freeze-dried (Christ, Alpha 1–4, Martin Christ Gefriertrocknungsanlagen GmbH, Osterode am Harz, Germany) for the determination of antioxidant activities and carotenoids. Another part was stored at −20 °C to determine volatile organic compounds (VOCs).

### 4.3. Determination of Leaf Color

At 33 DAT, leaf colorimetric indices were measured on 20 healthy and fully expanded leaves per replication (CR-400 portable colorimeter, Minolta Camera Co., Ltd., Osaka, Japan). The CIELab color space was used, where L, a*, and b* indicate the brightness and chromaticity of green and yellow, respectively. The Chroma and Hue angle were calculated according to Kheng [57] formulas.

### 4.4. Determination of Antioxidant Activities and Carotenoids

Two different spectrophotometric methods, DPPH (1,1-diphenyl-2-picrylhydrazyl) and FRAP (ferric reduction antioxidant power), were employed to determine basil antioxidant activities. For preparing the stock solutions and aqueous extracts, DPPH, and FRAP refer to the protocols of Brand-Williams et al. [58] and Rajurkar and Hande [59], respectively. Absorbances of the DPPH and FRAP assays were recorded with a UV-VIS spectrophotometer (Shimadzu, Japan) at wavelengths of 517 and 593 nm, respectively. Analyses were performed in triplicate and the results were expressed as mmol Trolox equivalents kg^–1^ of dry weight (dw).

The concentration of carotenoids (lutein and β-carotene) in basil leaves was determined in freeze dried samples by high performance liquid chromatography with diode array detection (HPLC-DAD) according to the protocol described by Ciriello et al [13]. Briefly, an aliquot (0.1 g) of lyophilized tissues was macerated in water and hexanol, sonicated, centrifuged, and solvent stripped. The resulting pellet, after two cycles of vacuum centrifugation, was mixed with methanol and MTBE (methyl-t-butyl ether) in a 1:1 ratio and analyzed with HPLC-DAD (LC 10; Shimadzu, Osaka, Japan) equipped with a 250 × 4.6-mm, 5-µm reverse-phase Gemini C18 column (Phenomenex, Torrance, CA, USA). Calibration curves were constructed using commercial β-carotene and lutein (Sigma-Aldrich, Milan, Italy), and the results were expressed in ppm dw. All analyses were performed in triplicate.

### 4.5. Determination of Aromatic Profile

The identification of volatile organic compounds (VOCs) was determined by gas chromatography coupled to a mass spectrometer (GC/MS; Agilent 6890 N; Agilent Technologies Italia, Milan, Italy) using a solid phase micro-extraction technique (SPME), as described in detail by Ciriello et al. [60]. Briefly, a divinylbenzene/carboxen/polydimethylsiloxane fiber (Supelco^®^, Bellefonte, PA, USA) was introduced into the headspace of the vial containing the previously heated sample (30 °C for 10 min) for the adsorption of VOCs and then inserted into the split-splitless injection system of GC/MS (250 °C; 7.64 PSI; flow rate 24 mL min^−1^; 10 min desorption step). The chromatographic run lasted 30 min and 67 s (temperature gradient mode 50–280 °C; helium as carrier gas with a flow rate of 1 mL min^−1^; mass spectrum at 70 eV). VOCs were identified by comparison with the NIST spectral database and expressed as a percentage of total area normalization. Each treatment was analyzed in triplicate.

### 4.6. Statistics

Data were subjected to analysis of variance (ANOVA) using IBM SPSS 28 software (International Business Machines Corporation, Armonk, NY, USA). One-way analysis of variance (ANOVA) and Student’s exact test were used to calculate the statistical significance of the mean effect of cultivars and salt stress, respectively. Two-way analysis of variance (ANOVA) was used to assess the significance of the effects of factor interactions. The statistical significance of the CV × S interaction and the CV factor was determined using the Tukey–Kramer HSD test at the level of *p* < 0.05 level. All data were presented as mean ± standard error. The principal component analysis (PCA) was performed with RStudio [RStudio Team (2021)] on groups of aromatic compounds.

## 5. Conclusions

Evaluating the response of the plant to abiotic limiting conditions, such as salinity, is a necessary step to understand the adequate tolerance of a horticultural species. The morphological and genetic differences of the basil plants evaluated in our experiment drove different responses to the salinity of NaCl (60 mM). Dark Opal and Italiano Classico enacted adaptive stress responses based mainly on the reduction in epigeal biomass, while in Purple Ruffles, the same saline conditions did not alter either yield or leaf color. However, the different behavior of the cultivars to salt stress was also confirmed by the nonunique changes in secondary metabolism. The flavor profile of the most susceptible cultivars (Dark Opal and Italiano Classico) showed more significant changes than that of Purple Ruffles. Although basil is repeatedly referred to in the literature as a moderately salinity-tolerant plant, this assumption is not universally valid because morphological variability is often overlooked. The different responses observed in our experiment suggest that tolerance to a given salinity is a function of genotype.

## Figures and Tables

**Table 1:** Mean effect of Cultivar (CV) and Stress (S) on shoot fresh weight (fw), leaves fresh weight (fw), and stem diameter of Dark Opal, Italiano Classico, and Purple Ruffles basil. Control: 0 mM NaCl; NaCl: 60 mM NaCl.

Treatment	Shoot fw	Leaves fw	Stem Diameter
	g Plant^−1^		mm
Cultivar (CV)			
Dark Opal	115.213 ± 10.047 b	107.716 ± 10.05 b	7.153 ± 0.487 b
Italiano Classico	168.49 ± 21.005 a	157.219 ± 19.822 a	9.274 ± 0.513 a
Purple Ruffles	60.306 ± 3.47 c	56.072 ± 3.248 c	6.293 ± 0.383 c
Significance	***	***	**
Stress (S)			
Control	135.657 ± 23.2 a	127.206 ± 21.746 a	8.043 ± 0.704 a
NaCl	93.682 ± 8.382 b	86.799 ± 7.85 b	7.104 ± 0.334 b
Significance	***	***	***

Data are mean values ± standard deviation, *n* = 3. ** and *** denote significant effects at *p* ≤ 0.01 and 0.001, respectively. Different letters within columns indicate significant mean differences according to Tukey HSD test (*p* = 0.05).

**Table 2:** Mean effect and interaction of Cultivar (CV) and Stress (S) on CIELab colorimetric parameters of Dark Opal, Italiano Classico, and Purple Ruffles basil. Control: 0 mM NaCl; NaCl: 60 mM NaCl.

Treatment	L	a*	b*	Chroma	Hue Angle
Cultivar (CV)					
Dark Opal	29.152 ± 1.139 b	4.038 ± 1.032 b	0.598 ± 1.564 c	6.195 ± 0.332 b	172.721 ± 9.28 b
Italiano Classico	42.725 ± 0.675 a	−15.232 ± 0.269 c	22.424 ± 0.569 a	27.127 ± 0.612 a	124.294 ± 0.317 c
Purple Ruffles	27.29 ± 0.507 b	6.168 ± 0.313 a	2.433 ± 0.574 b	6.892 ± 0.335 b	201.516 ± 4.996 a
Significance	***	***	***	***	***
Stress (S)					
Control	32.158 ± 2.801 b	−1.025 ± 3.617 a	7.077 ± 3.982 b	13.592 ± 3.509	157.122 ± 9.811 b
NaCl	33.953 ± 2.15 a	−2.326 ± 3.239 b	9.893 ± 3.061 a	13.217 ± 3.389	175.232 ± 13.542 a
Significance	**	**	***	n.s	***
CV × S					
Dark Opal × Control	26.698 ± 0.308 d	6.218 ± 0.241 a	−2.861 ± 0.089 c	6.86 ± 0.183	154.974 ± 1.615 b
Dark Opal × NaCl	31.606 ± 0.612 c	1.858 ± 0.718 b	4.057 ± 0.511 b	5.53 ± 0.274	190.467 ± 10.633 a
Italiano Classico × Control	43.271 ± 1.182 a	−15.479 ± 0.203 c	22.787 ± 0.871 a	27.58 ± 0.825	124.313 ± 0.688 c
Italiano Classico × NaCl	42.178 ± 0.762 b	−14.985 ± 0.511 c	22.061 ± 0.855 a	26.674 ± 0.993	124.275 ± 0.169 c
Purple Ruffles × Control	26.504 ± 0.144 d	6.185 ± 0.454 a	1.305 ± 0.142 b	6.336 ± 0.472	192.078 ± 0.738 a
Purple Ruffles × NaCl	28.075 ± 0.804 d	6.15 ± 0.531 a	3.561 ± 0.593 b	7.448 ± 0.175	210.954 ± 5.931 a
Significance	**	***	***	n.s	*

Data are mean values ± standard deviation, *n* = 3. n.s, *, **, and *** denote non-significant or significant effects at *p* ≤ 0.05, 0.01, and 0.001, respectively. Different letters within columns indicate significant mean differences according to Tukey HSD test (*p* = 0.05).

**Table 3:** Mean effect of Cultivar (CV) and Stress (S) on Lutein, β-carotene, DPPH, and FRAP antioxidant activities of Dark Opal, Italiano Classico, and Purple Ruffles basil. Control: 0 mM NaCl; NaCl: 60 mM NaCl.

Treatment	Lutein	β-Carotene	DPPH	FRAP
	ppm (dw)		mmol Trolox kg^−1^ (dw)	
Cultivar (CV)				
Dark Opal	836.335 ± 8.483 c	361.531 ± 10.468 c	173.563 ± 3.727 a	257.321 ± 5.148 b
Italiano Classico	885.376 ± 26.441 b	408.605 ± 5.406 b	99.347 ± 5.712 b	233.648 ± 5.643 c
Purple Ruffles	1122.581 ± 76.676 a	445.567 ± 28.932 a	173.374 ± 13.972 a	356.071 ± 5.989 a
Significance	***	***	***	***
Stress (S)				
Control	907.262 ± 20.381 b	394.964 ± 6.471 b	132.93 ± 11.527 b	270.13 ± 18.609 b
NaCl	988.932 ± 76.449 a	415.505 ± 25.18 a	164.593 ± 14.506 a	294.564 ± 18.932 a
Significance	***	***	***	***

Data are mean values ± standard deviation, *n* = 3. *** denote significant effects at *p* ≤ 0.001. Different letters within columns indicate significant mean differences according to Tukey HSD test (*p* = 0.05).

**Table 4:** Mean effect and interaction of Cultivar (CV) and Stress (S) on aromatic volatile compounds of Dark Opal, Italiano Classico, and Purple Ruffles basil. Control: 0 mM NaCl; NaCl: 60 mM NaCl.

Treatment	Aliphatic Aldeyde		Aliphatic Alcohol	Monoterpene Hydrocarbons			Aromatic Compounds		Sesquiterpene Hydrocarbons
	Hexenal	2-Hexanal	1-Octen-3-ol	Eucalyptol	β-cis-Ocimene	Linalool	Estragole	Eugenol	Trans-α-Bergamotene
		%							
Cultivar (CV)									
Dark Opal	4.034 ± 0.614 a	14.334 ± 1.273 a	2.374 ± 0.402 a	22.475 ± 2.183 a	1.359 ± 0.417 ab	30.083 ± 2.372 b	nd	3.246 ± 0.415 b	7.848 ± 1.957
Italiano Classico	1.583 ± 0.074 b	6.424 ± 1.313 b	1.276 ± 0.097 b	14.792 ± 0.722 b	2.39 ± 0.543 a	44.958 ± 2.161 a	nd	5.436 ± 0.214 a	4.418 ± 1.382
Purple Ruffles	nd	0.114 ± 0.023 c	0.513 ± 0.059 c	7.852 ± 1.156 c	0.015 ± 0.002 b	9.461 ± 1.097 c	79.499 ± 1.05	0.031 ± 0.005 c	2.772 ± 0.257
Significance	*	***	***	***	*	***		***	n.s
Stress (S)									
Control	nd	6.206 ± 2.261	1.689 ± 0.414 a	13.63 ± 2.157	0.803 ± 0.207 b	30.54 ± 6.119 a	80.509 ± 1.465	3.098 ± 0.795	5.607 ± 0.727
NaCl	2.809 ± 0.614	11.507 ± 1.545	1.373 ± 0.08 b	16.449 ± 2.555	2.552 ± 0.619 a	25.794 ± 4.347 b	78.49 ± 1.533	4.052 ± 0.748	5.242 ± 2.559
Significance	n.s	n.s	***	n.s	**	**	n.s	n.s	n.s
CV × S									
Dark Opal × Control	nd	nd	3.26 ± 0.133 a	19.497 ± 0.548 ab	1.191 ± 0.133 b	34.188 ± 0.15 b	nd	3.928 ± 0.128 ab	6.544 ± 0.035
Dark Opal × NaCl	nd	14.992 ± 0.709 a	1.488 ± 0.065 b	25.452 ± 3.828 a	1.528 ± 0.908 b	25.978 ± 3.356 c	nd	2.565 ± 0.617 b	9.152 ± 4.176
Italiano Classico × Control	4.034 ± 0.614	13.677 ± 2.677 a	1.295 ± 0.18 b	16.114 ± 0.561 bc	1.203 ± 0.169 b	49.596 ± 1.333 a	nd	5.334 ± 0.125 a	7.505 ± 0.122
Italiano Classico × NaCl	nd	3.511 ± 0.275 b	1.258 ± 0.119 b	13.469 ± 0.735 bc	3.577 ± 0.195 a	40.319 ± 0.222 b	nd	5.538 ± 0.45 a	1.331 ± 0.07
Purple Ruffles × Control	1.583 ± 0.074	9.338 ± 0.24 a	0.513 ± 0.059 b	5.278 ± 0.146 d	0.015 ± 0.002 b	7.837 ± 1.27 d	80.509 ± 1.465	0.031 ± 0.005 c	2.772 ± 0.257
Purple Ruffles × NaCl	nd	0.114 ± 0.023 b	nd	10.425 ± 0.205 cd	nd	11.085 ± 1.328 d	78.49 ± 1.533	nd	nd
Significance	n.s	*	***	*	*	**		*	n.s

Data are mean values ± standard deviation, *n* = 3. n.s, *, **, and *** denote non-significant or significant effects at *p* ≤ 0.05, 0.01, and 0.001, respectively. Different letters within columns indicate significant mean differences according to Tukey HSD test (*p* = 0.05). nd: not detected.

## Data Availability

The data are contained within the article.
